# Effects of nusinersen after one year of treatment in 123 children with SMA type 1 or 2: a French real-life observational study

**DOI:** 10.1186/s13023-020-01414-8

**Published:** 2020-06-12

**Authors:** Frédérique Audic, Marta Gomez Garcia de la Banda, Delphine Bernoux, Paola Ramirez-Garcia, Julien Durigneux, Christine Barnerias, Arnaud Isapof, Jean-Marie Cuisset, Claude Cances, Christian Richelme, Carole Vuillerot, Vincent Laugel, Juliette Ropars, Cécilia Altuzarra, Caroline Espil-Taris, Ulrike Walther-Louvier, Pascal Sabouraud, Mondher Chouchane, Catherine Vanhulle, Valérie Trommsdorff, Anne Pervillé, Hervé Testard, Emmanuelle Lagrue, Catherine Sarret, Anne-Laude Avice, Pierre Beze-Beyrie, Vanessa Pauly, Susana Quijano-Roy, Brigitte Chabrol, Isabelle Desguerre

**Affiliations:** 1 Centre de Référence des Maladies Neuromusculaires de l’enfant PACARARE, Service de Neuropédiatrie, Hôpital Timone Enfants, 264 rue Saint Pierre, 13385 Marseille Cedex 5, France; 2 Centre de Référence des Maladies Neuromusculaires Nord/Ile de France/Est, Hôpital Raymond Poincaré, APHP, Garches, France; 3 Centre de Référence des Maladies Neuromusculaires AOC, CHU d’Angers, Angers, France; 4 Centre de Référence des Maladies Neuromusculaires Nord/Ile de France/Est, Service de Neurologie pédiatrique, Hôpital Necker-Enfants Malades, APHP, Paris, France; 5 Centre de Référence des Maladies Neuromusculaires Nord/Ile de France/Est, Service de Neuropédiatrie, Hôpital Trousseau, APHP, Paris, France; 6 Centre de Référence des Maladies Neuromusculaires Nord/Ile de France/Est, Service de Neuropédiatrie, Hôpital Salengro CHU Lille, Lille, France; 7 Centre de Référence des Maladies Neuromusculaires AOC, Unité de Neurologie Pédiatrique, Hôpital des Enfants CHU Toulouse, Toulouse, France; 8 Centre de Référence des Maladies Neuromusculaires PACARARE, Hôpitaux Pédiatriques de Nice CHU – Lenval, Nice, France; 9 Centre de Référence des Maladies Neuromusculaires de l’enfant PACARARE, Service de MPR pédiatrique L’Escale Hôpital Femme Mère Enfant, Hospices Civils de Lyon, Bron, France; 10 Centre de Référence des Maladies Neuromusculaires Nord/Ile de France/Est, Pédiatrie médico-chirurgicale, CHU de Strasbourg - Hôpital de Hautepierre, Strasbourg, France; 11 Centre de Référence des Maladies Neuromusculaires AOC, Service de Pédiatrie, CHRU de Brest, Brest, France; 12 Centre de compétences des Maladies Neuromusculaires Nord/Ile de France/Est, Unité de Neuropédiatrie et médecine pédiatrique, Hôpital Minjoz, CHU de Besançon, Besançon, France; 13 Centre de Référence des Maladies Neuromusculaires AOC, Unité de Neurologie pédiatrique, CHU Pellegrin, Bordeaux, France; 14 Centre de Référence des Maladies Neuromusculaires AOC, Service de Neuropédiatrie CHU Montpellier, Montpellier, France; 15 Centre de Référence des Maladies Neuromusculaires Nord/Ile de France/Est, Site Reims enfant AMH, CHU Reims, Reims, France; 16 Centre de Compétence des Maladies Neuromusculaires Nord/Ile de France/Est, Service de pédiatrie 1, Hôpital d’Enfants, CHU Dijon Bourgogne, Dijon, France; 17 Centre de Compétence des Maladies Neuromusculaires Nord/Ile de France/Est, CHU de Rouen Charles Nicolle, Rouen, France; 18 Centre de Référence des Maladies Neuromusculaires PACARARE, Service de Pédiatrie, CHU La Réunion, Saint-Pierre, France; 19 Centre de Compétence des Maladies Neuromusculaires PACARARE, Service de Pédiatrie, CHU La Réunion, Saint-Denis, France; 20 Centre de Compétence des Maladies Neuromusculaires PACARARE, Neuropédiatrie, Clinique Universitaire Pédiatrique, Hôpital Couple Enfant – CHU Grenoble, Grenoble, France; 21 Centre de Compétence des Maladies Neuromusculaires AOC, Hôpital Clocheville, Service « Neuropédiatrie et Handicaps », Tours, France; 22 Centre de Référence des Maladies Neuromusculaires PACARARE, Centre hospitalo-universitaire de Clermont-Ferrand, Clermont-Ferrand, France; 23 Centre de Référence des Maladies Neuromusculaires Nord/Ile de France/Est, Nancy, Hôpital de Brabois, Vandœuvre-Lès, Nancy, France; 24 Service de Pédiatrie, Centre Hospitalier de Pau, Pau, France; 25 Centre d’études et de recherche sur les services de santé et la qualité de vie (CEReSS) EA 3279, Faculté de Médecine, Aix-Marseille Université, Marseille, France

**Keywords:** Spinal muscular atrophy type I, Spinal muscular atrophy type II, Nusinersen, Motor function measure, MFM

## Abstract

**Background:**

Spinal muscular atrophy (SMA) is an autosomal recessive neuromuscular disorder characterized by degeneration of the anterior horn cells of the spinal cord. Nusinersen has been covered by public healthcare in France since May 2017. The aim of this article is to report results after 1 year of treatment with intrathecal nusinersen in children with SMA types 1 and 2 in France. Comparisons between treatment onset (T0) and after 1 year of treatment (Y1) were made in terms of motor function and need for nutritional and ventilatory support. Motor development milestone achievements were evaluated using the modified Hammersmith Infant Neurologic Examination–Part 2 (HINE-2) for patients under 2 years of age and Motor Function Measure (MFM) scores for patients over 2 years of age.

**Results:**

Data on 204 SMA patients (type 1 or 2) were retrospectively collected from the 23 French centers for neuromuscular diseases. One hundred and twenty three patients had been treated for at least 1 year and were included, 34 of whom were classified as type 1 (10 as type 1a/b and 24 as type 1c) and 89 as type 2. *Survival motor Neuron 2* (*SMN2)* copy numbers were available for all but 6 patients.

Patients under 2 years of age (*n* = 30), had significantly higher HINE-2 scores at year 1 than at treatment onset but used more nutritional and ventilatory support. The 68 patients over 2 years of age evaluated with the Motor Function Measure test had significantly higher overall scores after 1 year, indicating that their motor function had improved. The scores were higher in the axial and proximal motor function (D2) and distal motor function (D3) parts of the MFM scale, but there was no significant difference for standing and transfer scores (D1). No child in either of the two groups achieved walking.

**Conclusion:**

Nusinersen offers life-changing benefits for children with SMA, particularly those with more severe forms of the disorder. Caregiver assessments are positive. Nevertheless, patients remain severely disabled and still require intensive support care. This new treatment raises new ethical challenges.

## Background

Spinal muscular atrophy (SMA) is an autosomal recessive neuromuscular disorder characterized by degeneration of the anterior horn cells of the spinal cord resulting in muscle atrophy and proximal muscle weakness without cognitive impairment. It is caused by a homozygous deletion or compound heterozygous alterations (mutation and deletion) in the *survival motor neuron 1* gene (*SMN1*) on chromosome 5q13. The centromeric copy of the gene (*SMN2*) produces transcripts of the SMN protein lacking exon 7 because of a C-to-T transition that creates an exon-splicing suppressor sequence. SMA phenotype severity depends on the amount of functional SMN protein produced [1] and seems to be related to the number of *SMN2* copies present [2, 3].

The clinical type of SMA is defined by the age of the onset and the severity of the disease, from type 0, with in utero onset and reduced or absent movement, muscle contracture and ventilatory support at birth, to type 4, with adult onset [4, 5]. SMA type 1 begins before 6 months of age, with generalized progressive muscle weakness and atrophy. Infants with SMA type 1 do not achieve independent sitting. Hypoventilation, poor cough strength and bulbar dysfunction lead to respiratory failure, and death usually occurs before 2 years of age without ventilatory support. Type 1 SMA can be separated into three subtypes: type 1a, in which head control is never achieved and signs appear in the neonatal period; type 1b, in which head control is never achieved but onset is after the neonatal period; and type 1c, in which head control is achieved and onset is after the neonatal period [6]. SMA type 2 begins after 6 months of age and patients can sit but are unable to walk. Multidisciplinary management including respiratory, nutritional and orthopedic care improves survival and long term outcomes in type 1 and 2. Patients with SMA type 3 can walk without support but scoliosis and loss of ambulation can occur.

A new treatment for SMA patients, nusinersen, has been available in France since May 2017. Nusinersen is an antisense oligonucleotide that acts as a splicing modifier targeting the intronic splicing silencer N1 in the *SMN2* intron and is delivered by repeated intrathecal injections. Nusinersen has shown some clinical efficacy in well-controlled clinical trials with prolonged survival after 2 years of age in different populations of SMA patients [7–10]. However, these motor benefits seem to be somewhat offset by an increased use of invasive treatments such as gastrostomy and ventilatory support [11, 12].

The aim of this article is to report the clinical outcomes related to the perception of children and caregivers with SMA types 1 and 2 in France after 1 year of treatment with intrathecal nusinersen.

## Methods

SMA patient files were provided by 23 French centers for rare pediatric diseases in the French FILNEMUS network for rare neuromuscular diseases. This multicenter study was commissioned by the neuromuscular commission of the French Society of Pediatric Neurology (Société Française de Neuropédiatrie), which holds monthly discussion group meetings. The data for this study were collected from May 2017 (when nusinersen became available in France) until February 2019. The inclusion criteria were:

1. A genetically proven *SMN1* mutation: homozygous deletion or compound heterozygous alterations (mutation and deletion) of exon 7 on chromosome 5q13
2. SMA type 1 or 2, as defined by the HAS (*Haute Autorité de Santé*, French National Authority for Health)
3. A complete clinical description at treatment onset (T0)
4. Treatment with intrathecal nusinersen injections for at least 1 year ±2 months (Y1). (Patients were evaluated after 10, 12 or 14 months’ treatment depending on the study center).

The data collected retrospectively were: date of birth, gender, age at diagnosis, SMA type, number of copies of the *SMN2* gene, age at treatment onset, motor development milestone achievement, nutritional and ventilatory support information before and after 12 ± 2 months of year of treatment.

In order to standardize care throughout the country, a standard form was made available for all centers. These forms were therefore filled at each visit by the local investigator. Forms were then anonymized.

Intrathecal nusinersen injections, adverse events, and evaluation by caregivers after one SMA type was defined using the Mercuri classification as 1a/b, 1c, or 2 [4].

Motor development milestone achievement was evaluated using different scales depending on the patient’s age at treatment onset.

The modified Hammersmith Infant Neurologic Examination–Part 2 (HINE-2) [13] and the Children’s Hospital of Philadelphia Infant Test of Neuromuscular Disorders (CHOP INTEND) [14] were used to assess the motor development of children less than 2 years old and the Motor Function Measure (MFM) was used for children older than 2 years (MFM20 for children between 2 and 5 years old and MFM32 for children above 6 years of age) [15]. The MFM scale assesses motor function in three domains: D1, standing and transfer; D2, axial and proximal motor function; and D3, distal motor function [16]. All MFM evaluations were carried out by an MFM trained and certified physiotherapist.

Nutritional support was defined as use of a nasogastric tube or gastrostomy and ventilatory support was defined as non-invasive or tracheostomy-assisted ventilation for more than 12 h per day.

Caregivers’ evaluations were graded using the Clinical Global Impressions-Improvement (CGI-I) scale [17] with 7 ratings scored from very much improved (rating 1) to very much worse (rating 7). The caregivers were the parents, and it was asked to one of them to fill the CGI-I scale.

Management and control of the data were performed by a clinical research associate using the Epidata® Software.

### Statistical analysis

Paired comparisons were performed to assess the evolution of the patients’ parameters after 1 year (± 2 months; initial visit versus after 1 year of treatment). Qualitative variables (such as the use of nutritional or ventilatory support) were compared using McNemar’s test and quantitative variables (such as the HINE-2 and CHOP INTEND scores) were compared using Wilcoxon signed rank tests for paired data. Results are presented as mean, median and range. Differences were considered statistically significant at *p* < 0.05.

The association between SMA Type and *SMN2* copy number was evaluated using a chi-squared test and the intensity of the association was evaluated using Cramér’s V.

The data were analyzed by age subgroup, under 2 years and above 2 years of age, with the second group subsequently split into two additional subgroups (2–5 years and 6–17 years).

Statistical analyses were carried out by a statistician using SPSS (IBM SPSS Statistics) version 20.0.

## Results

**Fig. 1:**
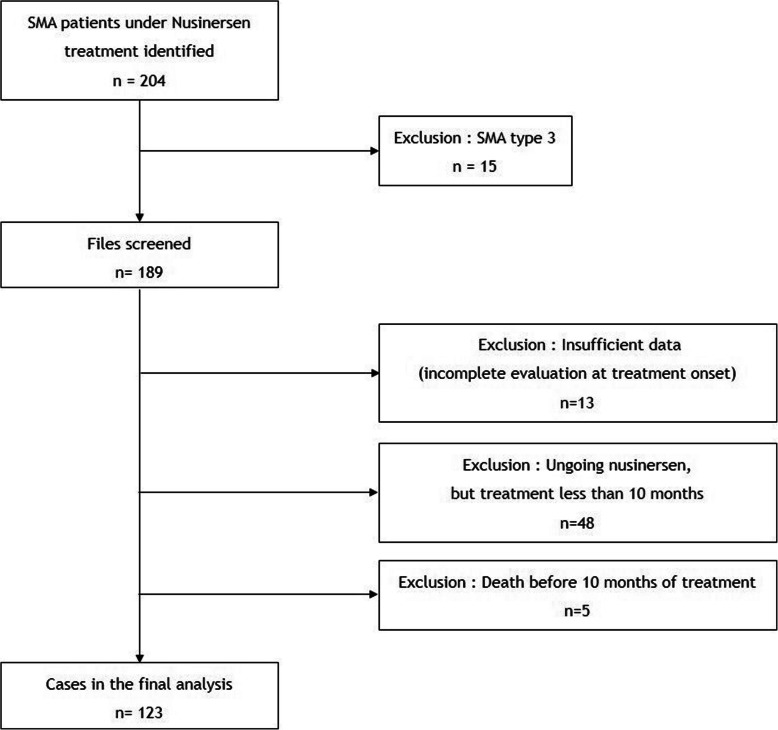
Flow diagram

Of the 204 SMA patients treated with nusinersen in the 23 French centers, 123 with at least 1 year of treatment were finally included in the study (Fig. 1). Five patients with SMA type 1a/b died before completing a full year of treatment and one patient with SMA type 1c died after 1 year of treatment.

### Population description

**Fig. 2:**
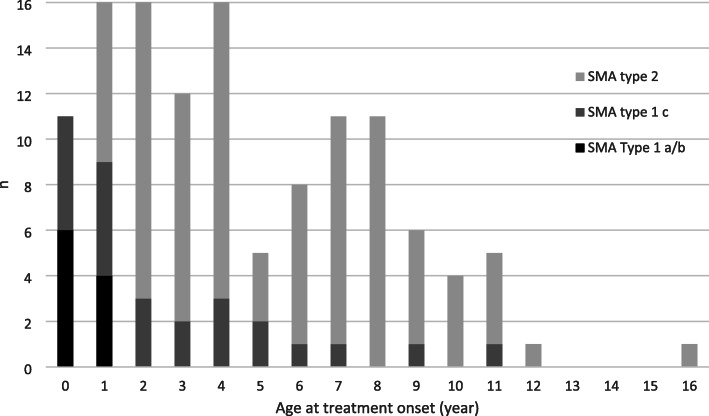
Patient numbers by age and SMA type

The patients’ characteristics are shown in Table 1. There were 68 girls (55.3%) and 55 boys (44.7%). Thirty-four patients were classified as type 1 (10 as type 1a/b and 24 as type 1c) and 89 as type 2. *SMN2* copy numbers were available for all but six patients: 18 patients had two *SMN2* copies, 96 had three *SMN2* copies, and 3 patients had four *SMN2* copies. There was a medium to strong association between SMA type and *SMN2* copy number (Cramér’s V = 0.33; 95% CI, 0.16–0.43, chisq test: *p* < 0.001) [18]. The patients’ age at treatment onset ranged from 3 months to 16 years (Fig. 2). Thirty children (24.4%) were under 2 years of age at treatment onset. Of the remaining 93 (75.6%) aged 2 years or older at treatment onset, 47 were 2–5 years of age and 46 were 6–17 years old.

**Table 1:** Patient characteristics

SMA type	Age at treatment onset				*SMN2 copy number*				
	< 2 y	2–5 y	6–17 y	Total	2	3	4	ND	Total
Type 1a/b	10	0	0	**10**	6	4	0	0	**10**
Type 1c	11	10	3	**24**	7	16	0	1	**24**
Type 2	9	37	43	**89**	5	76	3	5	**89**
**Total**	**30**	**47**	**46**	**123**	**18**	**96**	**3**	**6**	**123**

*ND* Not determined

One patient died after 14 months of treatment, of a viral cardiomyopathy.

### Need for nutritional and ventilatory support

**Fig. 3:**
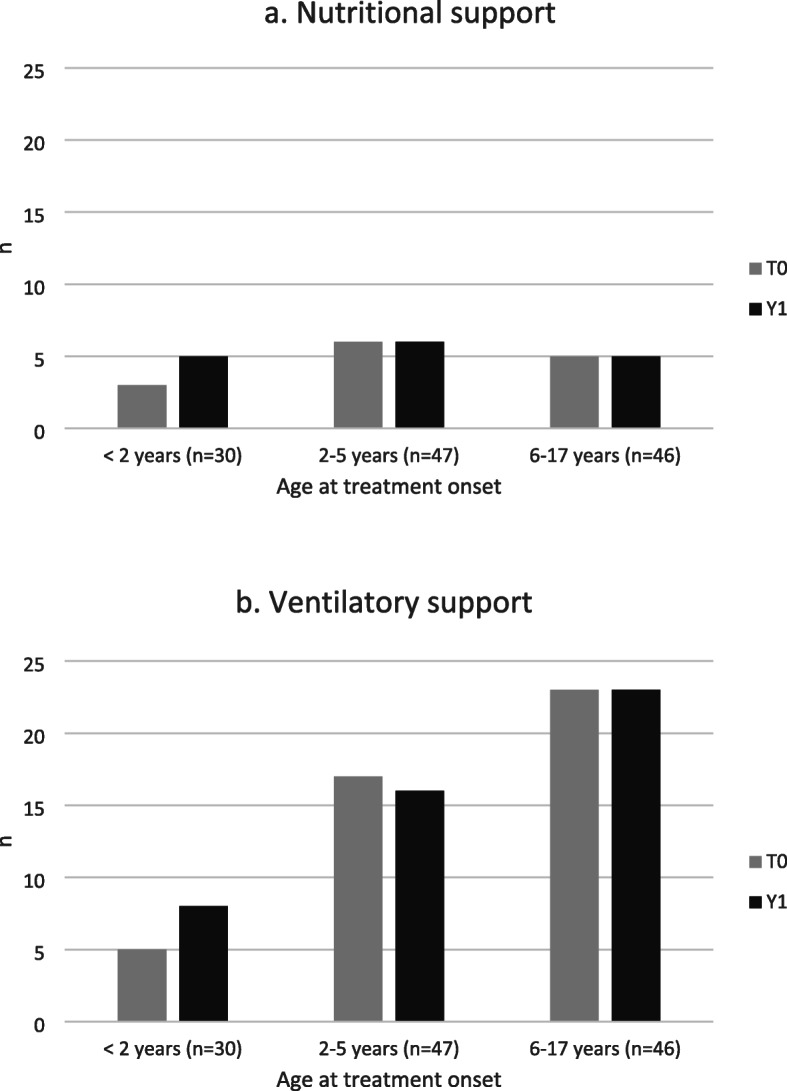
Number of patients requiring (**a**) nutritional and (**b**) ventilatory support at T0 and Y1

There was no significant change in the number of patients requiring ventilatory or nutritional support between T0 and Y1 overall (Fig. 3a and b). Among the youngest patients however (under 2 years old, SMA type 1), there was a non-significant increase in the number requiring nutritional support (three patients at T0 and five at Y1) and in the number requiring ventilatory support (five patients at T0 and eight at Y1).

### Motor function evaluation in children under 2 years of age (*n* = 30)

The patients’ motor function skills at T0 and Y1 were compared using the HINE-2 (20 patients with total scores only, 17 with detailed subscores) and/or CHOP-INTEND (*n* = 14) scales. Twelve patients were assessed using both methods, two patients were evaluated using MFM 20 only, and six patients were evaluated using different methods at T0 and Y1.

**Fig. 4:**
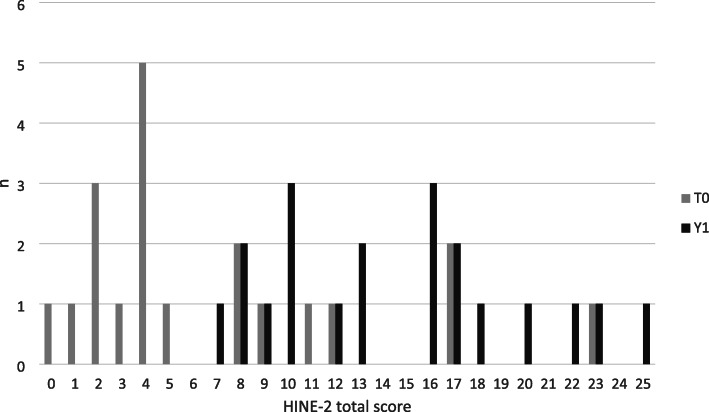
Distributions of HINE-2 scores at T0 and Y1 for patients aged less than 2 years

There was a statistically significant increase in the total HINE-2 scores between T0 and Y1 (*p* < 0.001). Analysis of the subscores revealed improvements in head control, sitting, voluntary grasp, ability to kick supine, rolling, crawling and standing milestones, but not for walking. Table 2 compares the patients’ HINE-2 motor function scores before and after 1 year of treatment. Figure 4 highlights the improvement in the total HINE scores after 1 year of treatment. The 14 patients who were assessed with the CHOP-INTEND scale also showed overall improvement, with the mean total score increasing from 35.1 to 50.3.

**Table 2:** Motor milestone (HINE-2) scores at T0 and Y1 for patients aged less than 2 years

HINE-2 score	T0	Y1	*P*
Head control (0–2) (*n* = 17)	1.2 / 1 (0–2)	1.9 / 2 (1–2)	**0.008**
Sitting (0–4) (*n* = 17)	1.4 / 1 (0–4)	2.8 / 3 (0–4)	**< 0.001**
Voluntary grasp – note side (0–3) (*n* = 17)	2.2 / 2 (0–3)	2.8 / 3 (2–3)	**0.008**
Ability to kick in supine (0–4) (*n* = 17)	1.4 / 1 (0–4)	3.2 / 4 (1–4)	**< 0.001**
Rolling (0–3) (*n* = 17)	0.6 / 0 (0–3)	1.8 / 2 (0–3)	**0.001**
Crawling or bottom shuffling (*n* = 17)	0.6 / 0 (0–4)	1.1 / 1 (0–4)	**0.008**
Standing (0–3) (*n* = 17)	0.1 / 0 (0–2)	0.6 / 0 (0–3)	**0.016**
Walking (0–3) (*n* = 17)	0.1 / 0 (0–1)	0.3 / 0 (0–2)	0.25
HINE- 2 Total score (0–26) (*n* = 20)	7 / 4 (0–23)	14.5 / 14.5 (7–25)	**< 0.001**

Minimum and maximum values for each sub-score are indicated in parentheses in the first column. The values shown are average / median (range). Statistically significant differences (*p* < 0.05) are highlighted in bold font

### Motor function evaluation in children older than 2 years (*n* = 93)

For these patients, motor function assessments at T0 and Y1 were mainly performed using the MFM scale (*n* = 68) (73% of the age subgroup; 5 with SMA type 1c and 63 with type 2 SMA) and only these data were analyzed. The 25 remaining patients were evaluated with either the HINE-2 or CHOP-INTEND methods.

**Fig. 5:**
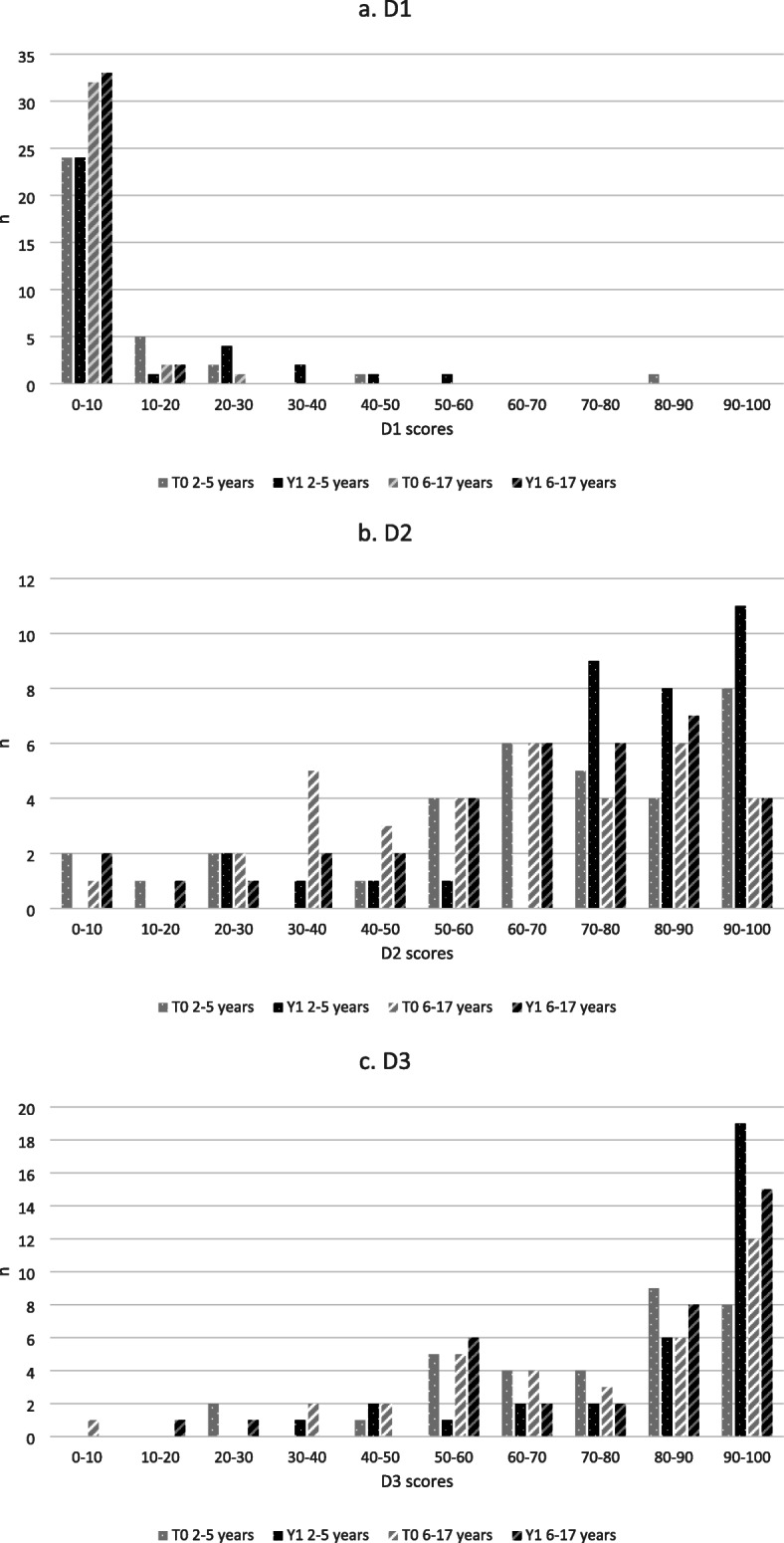
Distributions of scores in the three domains of the MFM at T0 and Y1

Table 3 compares the motor milestone achievements of the patients at T0 and Y1. Motor function improved slightly overall after 1 year of treatment (the median total MFM score increased by 6). In the different domains, there were significant improvements in the median scores in D2 (+ 9 percentage points, *p* < 0.001) and D3 (+ 7 percentage points, *p* < 0.001) but not in D1 (ambulation, − 1 percentage point). These improvements in D2 and D3 are clinically relevant in terms of functional benefits. In terms of age groups, the increases in MFM scores were more substantial in the children under 6 years old at treatment onset (*n* = 33, + 8 points overall, + 12 points in D2 and + 9 points in D3; *p* ≤ 0.001 in each case) than in those older than 6 years at treatment onset (+ 4 points overall, *p* = 0.099; + 6 points in D2, *p* = 0.457; and + 5 points in D3, *p* = 0.04). Figure 5 shows the distribution of MFM scores at T0 and Y1 for the D1, D2 and D3 domains.

**Table 3:** MFM scores at T0 and Y1 for patients older than 2 years

Age at treatment onset		T0	Y1	*P*
	MFM total score	42 / 44 (4–87)	47 / 50 (6–78)	**< 0.001**
All patients (*n* = 68)	MFM D1	7 / 4 (0–83)	7 / 3 (0–50)	0.245
	MFM D2	63 / 67 (1–100)	71 / 76 (2–100)	**< 0.001**
	MFM D3	74 / 81 (10–100)	81 / 88 (14–100)	**< 0.001**
2–5 years (*n* = 33)	MFM total score	45 / 45 (10–87)	52 / 53 (16–78)	**< 0.001**
	MFM D1	10 / 4 (0–83)	10 / 4 (0–50)	0.144
	MFM D2	66 / 71 (1–100)	77 / 83 (22–100)	**< 0.001**
	MFM D3	74 / 83 (24–100)	84 / 92 (38–100)	**0.001**
6–17 years (*n* = 35)	MFM total score	40 / 43 (4–60)	43 / 47 (6–63)	0.099
	MFM D1	4 / 3 (0–25)	3 / 3 (0–13)	0.651
	MFM D2	61 / 63 (6–96)	64 / 69 (2–97)	0.457
	MFM D3	73 / 81 (10–100)	79 / 86 (14–100)	**0.04**

The values shown are average / median (range). Statistically significant differences (*p* < 0.05) are highlighted in bold font

### General considerations

Caregiver evaluations of the benefits of treatment after 1 year were available for 105 patients. No caregiver rated their patient’s condition as minimally, much or very much worse (ratings 5, 6 and 7, respectively), 13% observed no change (rating 4), 35% a minimal improvement (rating 3), 46% considered that the patient’s condition was much improved (rating 2) and 6% very much improved (rating 1).

We counted 95 adverse events in 25 patients. They consisted in technical difficulties for lumbar puncture (*n* = 55) with fluoroscopic guidance required in 17 cases, headache (*n* = 23), post lumbar puncture syndrome (*n* = 6), nausea and vomiting (*n* = 4), asthenia (*n* = 4), back pain (*n* = 2), fever (*n* = 1).

Standard treatments such as motor and respiratory physiotherapy, orthoses (corset, brace) were maintained in keeping with recommended practice in France. Respiratory function was evaluated if possible but these data were not analyzed for this report.

## Discussion

In France, 204 children treated in referral centers for rare neuromuscular diseases received nusinersen between May 2017 (when the treatment became available) and February 2019, of whom more than 90% were patients with SMA type 1 or 2. This group of 204 patients represents about half of the children with SMA type 1 and type 2 in France. Based on ethical considerations and in accordance with parental wishes, some patients with SMA type 1 received palliative care without nusinersen [19]. In addition, some children and/or their parents refused the treatment because of the intrathecal delivery while in some older patients, intrathecal injections were impossible because of arthrodesis.

As already reported, the number of *SMN2* copies was found to be correlated with the patients’ phenotype. However, this correlation was only medium to strong and the phenotype could not be predicted based on the *SMN2* copy number alone, notably in patients with three copies of *SMN2*, in contrast with previous reports [3, 20]. Despite progress in understanding phenotype–genotype correlations in SMA, this issue remains to be clarified.

The benefits of nusinersen treatment were evaluated in the 123 patients who had received nusinersen for at least 1 year in the study period. No severe adverse events were observed, in agreement with previous studies [11, 12, 21, 22]. The results for this large cohort of patients with SMA type 1 (*n* = 34) and type 2 (*n* = 89) confirms that nusinersen improves motor function, in agreement with previous observational studies [11, 12, 21, 22]. Similar improvements have already been reported for children with type 1 SMA by Pechman et al. [11] in 61 patients followed for 6 months, by Aragon-Gawinska et al. in 33 patients followed for 6 months [12], and by Pane et al. in 85 patients followed for 1 year [21]. Darras et al.’s [22] is the only existing study to have reported on the effects of nusinersen treatment in patients with type 2 (*n* = 11) and type 3 (*n* = 17) SMA, who were followed for between 6 and 12 months. All but three of the children under 2 years of age in this study (27/30) had significantly improved HINE-2 scores after 1 year of treatment. However, our results confirm the importance of analyzing all the components of this score to properly evaluate the benefits of the treatment. Indeed, despite real improvements in motor function, none of the children were able to walk and the maximum motor level achieved seems to match that of a patient with type 2 SMA. Furthermore, our results confirm the previously observed [11, 12, 21] increased use of nutritional and ventilatory support. These treatments were in some case preventive to improve respiratory function and nutritional status.

The group of children older than 2 years (93 patients, 13 with SMA type 1c, 80 with SMA type 2), were evaluated using the French MFM test, which has never been used before in this context. The MFM assessments available for 68 patients indicated significant improvements in motor function, in keeping with those reported by Darras et al. [22] and Mercuri et al. [10]. Darras et al. [22] observed average gains for patients with SMA type 2 of 10 points on the Expanded Hammersmith Functional Motor Scale (HFMSE) and of 4 points on the Revised Upper Limb Module (RULM).) For our patients, there was no difference in the D1 domain (standing and transfer) scores, but an improvement in those for the D2 domain (axial and proximal motor functions, corresponding to the items of the HFMSE) and in those for the D3 domain (distal motor functions, corresponding to the RULM). The nutritional and ventilatory support requirements of this group of patients remained stable.

Interestingly, the improvements in motor function were greater in the subgroup of children younger than 6 years of age, who gained 12 points on average including 8 points in D3, whereas those aged 6–17 years only gained 6 points on average including 5 points in D3. In particular, 85% of the younger children had scores above 70% in D2, indicating that they could perform most normal daily activities (eating alone, combing their hair, rolling over during sleep...). However, no child in either of these two subgroups achieved walking. The motor benefit seems clearly related to early treatment as reported by other teams [12].

This is the first time that a clinical evaluation of a large cohort of SMA patients in a real life setting has been combined with an assessment of caregiver opinions (CGI-I scale). The positive responses of the caregivers despite generally mild motor benefits may reflect the considerable hopes invested in this new therapy, or on the hand, reflect limitations in the scales used to evaluate the benefits of the treatment. We did not use a generic tool for estimating the health-related quality of life of patients and/or caregivers. This should certainly be considered as a limitation of our study as this information is worth for the decision-making process, and it can offer the health benefits from a broader point of view not only for the patient also for the caregivers.

## Conclusions

Nusinersen changes the natural history and standard care of children with SMA [23, 24], particularly for severe forms and in younger children. It is a well-tolerated treatment. In spite of these improvements, patients with SMA types 1 and 2 treated with nusinersen still require intensive support care and remain severely disabled. There is nonetheless real optimism among caregivers after 1 year of treatment. This treatment raises new ethical challenges. We agree with King and Bishop [25] that families need to know more about what it means to live with SMA and what the benefits and burdens of the available treatments are. Parents of affected children and the patients themselves as they grow up face high stakes decisions about chronic treatment and potentially aggressive life prolonging therapies.

## Data Availability

The datasets used and analyzed during the current study are available from the corresponding author on reasonable request.
